# Usability of an eHealth sleep education intervention for university students

**DOI:** 10.1177/20552076241260480

**Published:** 2024-06-05

**Authors:** Lindsay Rosenberg, Gabrielle Rigney, Anastasija Jemcov, Derek van Voorst, Penny Corkum

**Affiliations:** 1 Department of Psychology & Neuroscience, Faculty of Science, 3688 Dalhousie University, Halifax, NS, Canada; 2 Appleton Institute, School of Health, Medical and Applied Sciences, Central Queensland University, Adelaide, South Australia, Australia; 3 Department of Psychiatry, Faculty of Medicine, 3688 Dalhousie University, Halifax, NS, Canada; 4 Department of Pediatrics, 3682 IWK Health Centre, Halifax, NS, Canada

**Keywords:** University students, insomnia, sleep health, digital intervention, usability testing

## Abstract

**Background:**

It has been estimated that more than one-third of university students suffer from insomnia. Few accessible eHealth sleep education programmes exist for university students and of the ones that do exist, fewer were developed using a user-centred approach, which allows for student input to be systematically collected and utilized to provide students with a programme that they consider to be easy to use and implement and to be effective. *Better Nights, Better Days-Youth* (*BNBD-Youth*) is a four-session eHealth sleep education programme designed for youth but previously only evaluated in younger adolescents (ages 14–18 years).

**Aims:**

The purpose of this study is to evaluate the usability of the *BNBD-Youth* programme with university students using Morville's User Experience Honeycomb framework to determine if this programme would meet the needs of university students and if so what modifications would be needed.

**Methods:**

Canadian undergraduate students (*n* = 46) completed the *BNBD-Youth* programme. Students completed online usability questionnaires based on the seven dimensions of Morville's User Experience Honeycomb (i.e. useful, usable, valuable, credible, desirable, accessible and findable) after each session and after completion of the programme. Open- and closed-ended questions were used to obtain both quantitative and qualitative responses.

**Results:**

Average quantitative ratings were positive across user experience dimensions, ranging from 3.43 to 4.46 (out of 5). Qualitative responses indicated overall positive experiences with the programme. The only constructive feedback that met the criteria for revising the programme was to include more interactive features in Session 4.

**Conclusions:**

This study demonstrates that university students found *BNBD-Youth* to be a usable programme for older youth. Demonstrating usability is an essential step in developing a programme with a user-centred design that university students will want to use in the future. Once the *BNBD-Youth* programme is revised to create the BNBD-University (*BNBD-Uni*) programme, additional usability and effectiveness testing will be conducted.

## Introduction

Many university students suffer from poor sleep health. An American multi-university study of over 7000 students found that 64% met criteria for poor sleep.^1^ The National Sleep Foundation recommends that young adults need 7 to 9 hours of sleep to function at their best^2^; however, many university students are not meeting this recommendation. Specifically, among university students, prevalence rates range from 9.4% to 38.2% for insomnia. Insomnia is defined as chronic and specific difficulty falling asleep, staying asleep and/or early morning awakenings despite adequate opportunity for sleep and associated with distress or impairment in functioning.^3,4^

Many factors contribute to insomnia (defined henceforth as those meeting either the full symptom range, or partial symptoms) in university students. During adolescence, there is a biological shift in circadian preference for later sleep and wake times. Bedtimes and wake times become progressively later, reaching a maximum at around 20 years of age.^5^ Furthermore, many psychosocial factors have been found to interfere with sleep, including waking up at night due to noise of other students when living in shared accommodations, bedtimes and waketimes on weekends and weekdays that frequently differ more than 1 to 2 hours and socializing late in the evening.^6,7^ In addition, many studies have demonstrated the negative impact of Internet usage and social media use at bedtime on sleep outcomes.^8,9^

Insomnia can negatively impact functioning across many aspects of daily living. Insomnia is associated with mental health consequences such as anxiety and depressive symptoms.^10–12^ Furthermore, chronic insomnia (insomnia lasting at least three months) has been demonstrated to be associated with increased stress, fatigue, lower quality of life and higher rates of substance use for sleep problems.^12^ Given the wide-ranging negative consequences of insomnia, accessible interventions targeting sleep and healthy sleep practices are of paramount importance. Within the broad spectrum of sleep interventions, sleep education interventions (e.g. in-person lectures, educational sessions on healthy sleep practices) are effective in improving sleep outcomes.^13,14^ Despite there being several effective university sleep education interventions, the majority are not easily accessible and frequently require in-person sessions that can be difficult to accommodate in students’ schedules. One way to bridge the accessibility gap is to use eHealth (i.e. technology applied using the internet, in which healthcare services are provided to improve quality of life outcomes and facilitate healthcare delivery).^15^ Some of the potential benefits of eHealth delivery methods include being able to reach a wider number of individuals who require services, scalability of the programme, affordability of services and flexibility for the individual.^16^ To this end, there has been an increasing focus on developing digital interventions to address sleep problems, as this would enhance accessibility.

Several reviews have been conducted to evaluate the use and usability of eHealth apps focused on sleep across the lifespan. A scoping review conducted by Bullock et al., 2022 on mobile phone applications for shift workers found only two papers met inclusion criteria, suggesting a dearth of studies that highlight sleep management apps for this population.^17^ A literature review by Rowan et al., 2024, evaluated the evidence for efficacy and quality of CBT-I sleep apps for ages 12 and older. The authors found the most commonly used components being sleep hygiene and relaxation/meditation. They found the strongest evidence for success was stimulus control and sleep restriction. In their review they found the most efficacy with studies that used self-report measures of sleep, with more mixed results on measures of sleep efficiency taken from objective measures of sleep and sleep diaries.^18^ In a review conducted by Nuo et al., 2023, on sleep apps, it was found that the largest proportion of sleep apps focused on sleep-tracking, followed by apps that intervene with sleep and apps that diagnose sleep issues. The majority of apps focused on breathing-related sleep disorders and insomnia sleep disorders. The study additionally indicated that although many sleep apps have been developed, most of the newer apps are not being used in the real world, but rather being used in laboratory studies. The review suggests that this may be attributed to app usability, suggesting that apps with high usability will be more likely to be used and recommended to others.^19^

There have been several empirical studies evaluating eHealth sleep interventions for adolescents and young adults. Consistently, these studies have found the programmes to be of high acceptability^20–23^ and have proven to be effective at improving sleep duration, sleep difficulties and sleep knowledge.^24^ An example is a study on the feasibility and acceptability of the DOZE eHealth programme for improving sleep for youth ages 15–24 years found the programme to be highly acceptable in areas including ease of use, understandability, time commitment and overall satisfaction. The programme was also rated as credible to participants.^23^ An experimental study conducted by Chu et al. (2018) evaluating a mobile sleep-management learning system for university students found the programme was easy to use and useful for students, with the number of students having decreased insomnia. Additionally, they found significant improvements in morning lingering in bed, total wake time, sleep efficiency, total sleep time and self-reported insomnia severity. They additionally found significant improvements in anxiety, depression and energy.^25^

Several eHealth sleep interventions for university students have been developed and studied^25–28^ However, few studies have demonstrated improvements in sleep outcomes.^25,28,29^ Furthermore, of the limited literature that exists, very few studies reported incorporating end-users in programme development.^30–32^ User-centred designs incorporate user-activities and user-feedback throughout the development process as guidance toward the needs of end-users.^33,34^ Research has demonstrated that involving end-users can reduce the development time and increase user acceptance.^35,36^ Understanding user acceptance is critical, as it has been demonstrated that a quarter of all online application downloads are only used one time.^37,38^ This underscores the importance of creating programmes that maintain user engagement. It has also been demonstrated that using an iterative process, whereby usability is tested early in development can allow for identification of important requirements for end-users that elicit behaviour change, and the identification of undesirable aspects of a programme that hinder a programme's adoption.^39^ Furthermore, it has been recommended that eHealth or telehealth programmes use end-user participation in development in order to produce programmes that are better tailored to user needs.^40^ In addition, in a survey of practitioners who have used user-centred design formats for at least three years, the majority reported that using user-centred design methods significantly improved the usefulness (79%) and usability (82%) of products developed in their organizations.^41^ This improved usability and usefulness ultimately may lead to higher rates of programme retention.

Considering what is known about the benefits of user-centred designs, it is surprising that very few eHealth sleep education intervention studies for university students have reported on end user inclusion in the development and evaluation process.^30–32,42^ One study that evaluated the effectiveness of an email intervention reported using feedback from users only to guide future iterations, rather than the current iteration of the programme.^32^ Furthermore, the researchers only asked participants about their perceptions of effectiveness of the strategies to use, and what could be changed to improve upon them. Another eHealth study reported using focus groups to inform the development of a text-message intervention to allow for refinement before testing the effectiveness of the intervention, however no information was reported on the results of the discussion.^32^ Additionally, no individual tailoring was reported in this intervention, with students only receiving standardized text messages with sleep-related educational content. Results of effectiveness testing demonstrated no significant differences on sleep quality, sleep hygiene behaviours, or sleep knowledge.

A third eHealth sleep education intervention was an integrated intervention for heavy alcohol use and sleep difficulties.^30^ The intervention was an online programme that delivered evidence-based sleep content, behavioural advice, relaxation training and cognitive strategies to target maladaptive beliefs about sleep. Focus groups were used to guide development of the programme, with 24 college students giving their perceptions of sleep and alcohol/sleep interactions and their behavioural health treatment preferences.^30^ Results demonstrated an overall positive reaction to having an integrated alcohol use/sleep intervention and the majority wanted the content to be personalized to their unique health profile. Participants also requested easy navigation, a way to monitor progress, daily added content and the option for peer or clinician support. A second set of focus groups were utilized with a beta-model of the programme to gain feedback through watching a power-point, however no results were reported. The study incorporated feedback by delivering new health information modules each week and giving a personalized summary of health characteristics from students’ initial questionnaire data (e.g. sleep quality, alcohol use), however, did not tailor the recommendations in the programme to their sleep data. Results of pilot testing demonstrated effectiveness in improving sleep quality and sleep-related impairment ratings.^31^

Despite the limited use of user-centred design in the development and evaluation of eHealth sleep interventions for university students, end-user engagement has been often employed in other areas of paediatric behavioural health. For example, King et al.^43^ engaged children, parents and educators in their evaluation of eHealth intervention for chronic pain, McManama O’Brien et al.^44^ engaged adolescents and their parents when developing a smartphone app for suicidal adolescents and Nitsch et al.^45^ engaged youth with body image or disordered eating symptoms to evaluate an online intervention. A range of methodologies were used including think-aloud activities (i.e. sharing thoughts as one uses the programme), demonstration and discussion, as well as semi-structured interviews and surveys/questionnaires where the participants are asked to reflect on their experiences with the programme, resulting in both quantitative and qualitative data. The results of these studies indicated needed changes in terms of delivery (e.g. make easier to navigate) and content (e.g. reducing content to main points).

Given the limited research on sleep interventions to be used with university students, there is a clear need for more accessible eHealth sleep education interventions that were developed utilizing a user-centred design. Our team previously developed and evaluated the usability of eHealth sleep education programme, *Better Nights, Better Days-Youth* (*BNBD-Youth*). The *BNBD-Youth* programme provides education about healthy sleep practices to improve behaviours and outcomes involving sleep in youth. The development of *BNBD-Youth* incorporated ideas from adolescents via focus groups, with adolescents requesting a programme that was interactive in which they can spend short periods of time engaging with the programme.^46^ Taking this into consideration, *BNBD-Youth* was designed using a micro-learning format, with small, digestible lessons. It provides psychoeducation, strategies for healthy sleep practices and behavioural sleep recommendations to improve sleep, as well as individualized sleep diaries to help with sleep tracking and habits, which is consistent with the extant literature on sleep programme design.^47,48^

The *BNBD-Youth* eHealth programme has been tested for usability and effectiveness with youth ages 14–18 years (papers under review). Usability testing results were positive, suggesting that the programme required only minimal modification (e.g. making the programme more desirable by adding more features, less text and improved visual design and improved compatibility for interactive features and videos on various devices).^49^ Once these revisions were made, a pilot effectiveness study was conducted. Results found that based on a pre-post design the intervention resulted in significant improvements on sleep variables including the sleep efficiency, total sleep time, total time in bed, number of night wakings, perception of wake-up time, perception of sleep quality and adherence to healthy sleep habits for the younger adolescent group.^50^

Dr. Corkum and her colleagues have also developed and evaluated a separate, unique eHealth programme for parents of children 4–12 with neurodevelopmental disorders.^51–53^ In a study that examined barriers and facilitators of using the programme using semi-structured interviews with parents (*n* = 15), many facilitators were established including increased self efficacy, positive outcomes for the family, improved sleep-related beliefs and attitudes and increased motivation. Barriers parents faced included challenges around time, difficulties implementing the strategies to try to improve sleep and psychosocial factors that led to challenges with implementation (e.g. burnout, stress and exhaustion, difficulty for family to adapt to intervention strategies).^53^ A randomized controlled trial (RCT) was conducted to evaluate the programme effectiveness and preliminary results demonstrated parental reports of effectiveness, parent satisfaction and implementation success.^52^

The goal of the current study is to gain feedback from older adolescents (i.e. university students ages 18–24 years) to determine if the original intervention (*BNBD-Youth*) could be used by older adolescents, and if so, what changes would be needed to be made to the programme. As many university students have insomnia,^4^ and many factors specific to university students contribute to inadequate sleep (e.g. more socializing late at night, waking up at night due to noise of other students in shared living accommodations),^6,7^ it is important to evaluate whether the original *BNBD-Youth* programme can be helpful for university students to learn more about sleep, understand their own sleep habits and learn strategies to improve upon their sleep, while limiting barriers to accessibility.

As such, the primary aim of the present study is to evaluate, using the User Experience Honeycomb framework,^43^ the usability of *BNBD-Youth* with university students ages 18–24 and to collect information about how best to modify the programme to the needs of university students with insomnia and/or symptoms thereof, creating *Better Nights, Better Days* – *University* (here forth called *BNBD-Uni*). The User Experience Honeycomb framework^54^ includes seven dimensions – usability, usefulness, accessibility, credibility, desirability, valuableness and findability. All of these dimensions impact desire and appeal to use a programme and motivation to continue using a programme. The information gained from this study will be used to modify the *BNBD-Youth* programme to make the *BNBD-Uni* programme, which then will be further tested for usability and effectiveness.

## Materials and methods

### Intervention

In the original *BNBD-Youth* version, which was used for the current study, users are emailed a link to the programme, with an included username and password. Upon clicking the link, they are taken to the programme log in page where they enter their username and password. Upon logging in, they are taken to the homepage with a tutorial icon that leads to a video tutorial of how the programme works. By clicking each session icon, the user is directed to the session content (see Table 1 for session content). There is an additional icon for accessing the electronic sleep diary, in which participants can enter information about their sleep each night, with the programme creating graphs to show various sleep variables. A final icon can be clicked to display all the personal answers users type into the programme to provide them their individualized sleep goals and plan.

**Table 1.** Better nights, better days programme content.

Lesson	Micro-learning content
*Session 1*	*What is Sleep*
1	Facts and fiction about sleep
2	What sleep is?
3	The sleep cycle and stages of sleep
4	Regulation of sleep
5	Are you a morning, or night person?
6	How sleep changes in adolescence
7	Sleep duration recommendations
8	Sleep and mental health
9	Relationship between sleep and mental health
10	What happens in the body when we do not get enough sleep
*Session 2*	*Your Sleep*
1	Important variables for measuring sleep
2	Understanding sleep diaries, starting to track your own sleep
3	Different ways to measure sleep
4	Common sleep disorders
5	Understanding and measuring sleep quality
6	Understanding and measuring healthy sleep practices
7	Bedtimes and waketimes
*Session 3*	*Healthy Sleep Practices*
1	The ABCs of SLEEPING
2	**A**ge-appropriate **B**edtimes and waketimes, with **C**onsistency
3	**S**chedules and routines
4	**L**ocation
5	**E**lectronics
6	**E**xercise and diet
7	**P**ositivity toward sleeping, going to bed relaxed
8	**I**ndependence
9	**N**eeds met during the day
10	**G**reat sleep
*Session 4*	*Looking Forward*
1	Checking in
2	Troubleshooting roadblocks
3	Extra Resources
4	Congratulations

*BNBD-Youth* includes four sessions which: (a) focuses on understanding sleep and its importance, (b) provides information on various sleep measures and sleep disorders, as well as learning about one's own sleep quantity and quality, (c) enhances their user's understand of the importance of healthy sleep practices and (d) provides for planning and preparing for healthy sleep (see Table 1 for a description of the micro-lessons).

### Participants

Participants were recruited through word of mouth from the researchers, posters placed around the Dalhousie University campus, and through online methods including the Facebook, Kijiji and Twitter. The target audience was university students across Canada who experience insomnia or symptoms of insomnia. Individuals were eligible if they were 18 to 24 years old, experienced symptoms of insomnia in the subthreshold range, attended a university, resided in Canada, had access to the Internet and e-mail, had no hearing or cognitive difficulties that they thought would interfere with participation and could understand and speak English. Participants were eligible to receive up to $60.00 in Amazon.ca gift cards for their participation in the study. Study recruitment spanned March 2019 to May, 2020.

### Procedure

Interested participants were directed to the online advertisement. Those who viewed the online advertisement clicked a link that directed them to the online Eligibility Questionnaire via the secure survey software, Opinio. Participants who met inclusion/exclusion criteria were then e-mailed an online Information and Consent form. Upon providing consent, participants were e-mailed a questionnaire to collect data on demographic information and sleep behaviours and practices. After these questionnaires were completed, participants were given access to the *BNBD-Youth* programme. Participation was monitored by one of the researchers, whereby a usability questionnaire was sent out prior to completing each of four sessions, to allow for participants to complete the questionnaire as they moved through the session and after completion of the programme. Questionnaires were only sent out after completion of the prior session and questionnaire occurred, to ensure participation in each session. Participants had a maximum of five weeks to complete the programme. Participants were asked to complete the sessions at a rate of one session per week, with an additional week incorporated between Sessions 2 and 3. Sessions were expected to take approximately 30–45 minutes each to complete and participants could exit and return to sessions to complete them at any time. Reminder e-mails were sent if participants had not completed the questionnaire before beginning the next session. This study was approved by the IWK Research Ethics Board (#IWK REB File Number: 1023977).

### Measures

#### Eligibility questionnaire

The questionnaire was created by the study authors for a previous study,^49^ and asked whether participants were 18–24 years of age, have no hearing or cognitive deficits that would interfere with participation, have access to the internet and an e-mail account and can understand and speak English.

#### Insomnia severity index (ISI)

The ISI questionnaire is a measure used to detect insomnia. The ISI includes 7 items rated on a 5-point Likert scale (0 = no problem; 4 = very severe problem).^55^ Scores on the ISI range from 0 to 28, with 8 to 14 considered to be subthreshold insomnia symptoms, 15 to 21 moderate symptoms and 22 to 28 severe symptoms. The ISI demonstrates a high internal consistency in adults (*α *= 0.90; Morin et al., 2011) and adolescents (*α *= 0.90).^56^ The ISI was used to determine if the participant met inclusion criteria of, at minimum, subthreshold insomnia.

#### Demographic questionnaire

The questionnaire was created by the study authors and included questions regarding the participant's age, sex, university attended, whether an international student, location, living arrangement (i.e. living at home with family, in residence, alone in the community, with housemates in the community), ethnic/cultural heritage, highest level of educational attainment and employment status.

#### Sleep hygiene index (SHI)

The SHI is a 13-item questionnaire that asks about current sleep behaviours and practices.^57^ The SHI requires participants to report how frequently they engage in certain poor sleep practices, such as taking naps, exercising before going to bed, worrying in bed and caffeine and alcohol intake, on a 5-point scale from ‘Never’ to ‘Always’. The scores are then summed to produce a global sleep hygiene score ranging from 13 to 65, in which higher scores indicate poorer sleep hygiene. The SHI has demonstrated moderate internal consistency (*α* = 0.66).

#### Usability questionnaires

The Session Feedback Questionnaire contained 33 items used to assess usability for each of four *BNBD-Youth* sessions, with a range of between 1 and 7 additional questions specific to each session (e.g. The ‘Are you a Lark or an Owl’ quiz helped me to better understand natural sleep timing). The questionnaire was estimated to take approximately 15 minutes. The Programme Feedback Questionnaire contained 50 questions used to evaluate usability of the programme in its entirety. The Programme Feedback Questionnaire was estimated to take approximately 25 minutes to complete. Both questionnaires included questions based on Morville's User Experience Honeycomb. Morville's User Experience Honeycomb was used as it was designed to evaluate usability of online platforms and has been used for evaluating the usability of several online websites, programmes and applications.^58–60^ Questionnaires were adapted from prior usability studies evaluating interventions around sleep.^49,61,62^ Open- and closed-ended questions regarding each of Morville's user experience dimensions were presented, including questions as to whether the session or programme was usable (e.g. user-friendly, appropriate length), useful (e.g. helpful for understanding sleep needs), findable (e.g. information easy to find), desirable (e.g. programme desirable to go through, information what they were looking for), valuable (e.g. meeting their goals), credible (e.g. information trustworthy) and accessible (e.g. would all users be able to use the programme). For closed-ended questions, a 5-point Likert scale was used to rate agreement on statement (1 = strongly disagree, 3 = neither agree nor disagree, 5 = strongly agree), with open text boxes for participants to elaborate on their responses for each user experience dimension. Ratings of 1 to 2 were considered negative, 3 was considered neutral and ratings of 4 to 5 were considered positive. The session questionnaires additionally asked about favourite and least favourite features, most and least engaging aspects of each session/programme and whether information should be added, removed or whether the order of information should be changed. The Programme Feedback Questionnaire additionally asked specific closed-ended questions about whether participants liked and/or used certain aspects of the programme (e.g. sleep diary, videos, interactive features, reminders to complete the sleep diary), technology (e.g. whether the programme worked on their device, which device they used to access the programme), type of OS system used) and general feedback questions (e.g. overall satisfaction, whether information should be removed, added, reordered) (See Supplementary Material).

### Data analysis

Descriptive statistics were used to analyse the data from the demographic questionnaire, including frequency counts, means, standard deviations and percentages. The SHI and ISI questionnaires were summed by individual and then averaged across individuals.

Closed-ended questions (i.e. quantitative data) were analysed by using descriptive statistics including means, standard deviations and ranges for each of the Session Feedback Questionnaires and the Programme Feedback Questionnaire. Each usability dimension included two closed ended questions that were averaged for each participant to obtain a score for each dimension (see Supplementary Materials for the questions each dimension was derived from).

Open-ended questions (i.e. qualitative information) were analysed and coded using directed content analysis, which allows coding to be made within an existing framework. Following this, a second round of coding was completing to identify themes within each of the user experience categories. Two coders independently coded the comments to the User Experience Honeycomb categories and one coder coded themes within these categories, with the second coder coding according to the themes independently. Inter-rater reliability was calculated using Cohen's kappa (κ =0.93) and disagreements were discussed until consensus was obtained. Finally, within each dimension, all suggestions and constructive feedback were tallied based on the number of participants who endorsed the suggestion for each session questionnaire and the programme questionnaire. Comments within each questionnaire that countered these suggestions were tallied and a percentage in support of change was created. As used in prior usability studies, a minimum criterion of 10% in support of change was required to consider making the change.^47^ For example, if there were 100 participants and 20 stated they disliked the visuals and 5 endorsed enjoying the visuals, a percentage change of 15% would be reported, meeting the criterion of 10% required to consider making a change to the programme.

## Results

### Participants

**Figure 1.**
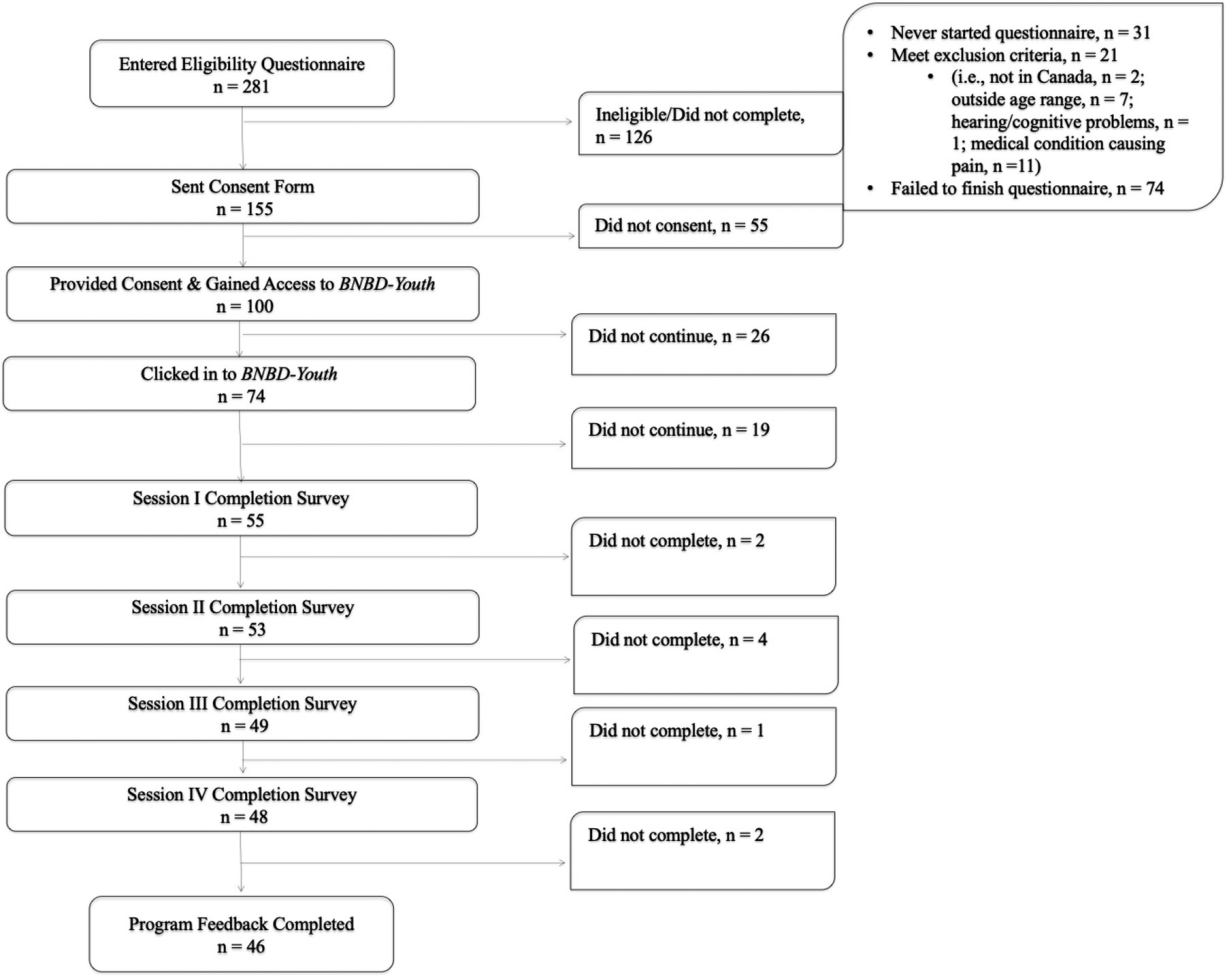
Participant flow diagram depicting the number of participants at each stage of study progression.

Of 85 participants who consented to the study and completed the demographic questionnaire, 53 participants gained access to the programme and completed at least one questionnaire. Of these 53 participants, 46 completed all questionnaires and were included in the analysis (see Figure 1). Given this was a usability study, it was important that the feedback used in the study was from those who had experienced the programme in its entirety, to allow consideration of all aspects of the programme.

The 46 participants who completed all questionnaires had a mean age of 20.6 years (*SD *= 1.6, range = 18–24). The participants were largely Caucasian (56.5%) and closely distributed on sex (54.3% female). Most participants were Canadian (56.5%), although a sizable number were international students (43.5%). Participants lived across five Canadian provinces with the majority living in Ontario (60.9%) and most participants living in a city over 500,00 people (60.9%). Participants described attending a variety of universities (*n* = 18 universities), with the largest percentage (17.4%) attending the university where the research was conducted. The majority of participants reported living with housemates in the community (56.5%). Most participants reported their highest level of education as some university (32.6%) or completed high school education or equivalent (28.3%). The overwhelming majority of participants reported employment status as ‘student’ (95.7%). Across participants, scores on the Insomnia Severity Index ranged from 12 to 27, with a mean of 21.4 (SD = 3.3), falling between the moderate and severe range. Internal consistency was demonstrated to be in the adequate range (α = 0.70). Participants’ sleep habits as reported on the Sleep Hygiene Index, had a mean score of 32.3 (SD = 5.7), falling within the upper half of the scale, where higher scores indicate poorer sleep practices. The Sleep Hygiene Index demonstrated moderate internal consistency (α = 0.60).

### Usability ratings

The following results are reported separately for the overall programme ratings and the session ratings, with overall programme results first, followed by session ratings.

#### Overall programme ratings

##### Quantitative analyses

Mean usability ratings for the overall programme on Morville's user experience dimensions ranged from 4.03–4.46 on a scale of 1–5 (1 = strongly disagree, 3 = neither agree nor disagree, 5 = strongly agree). Of the seven ratings, no mean ratings from the overall programme were identified as negative (score of 1 or 2) or neutral (score of 3). All mean ratings were identified as positive (4–5) (see Table 2).

**Table 2.** Overall programme ratings.

	Overall programme ratings (*n* = 46)
Useful	4.46 (0.43)
Usable	4.03 (0.69)
Findable	4.23 (0.69)
Desirable	4.35 (0.48)
Accessible	4.33 (0.46)
Credible	4.37 (0.44)
Valuable	4.41 (0.46)

*Note*. Rating scale from 1 to 5 (1 = strongly disagree; 3 = neither agree nor disagree; 5 = strongly agree). Dimensions were derived from Morville's user experience honeycomb (Morville & Sullenger, 2010).

##### Qualitative analyses

For the dimension of usefulness, participants provided only positive comments with 73.9% of participants (*n* = 34) noting that the programme was helpful to better understand and manage healthy sleep practices (see Table 3 for example feedback from participants).

For four dimensions, there was more positive comments than constructive comments. For findability, 65.2% (*n* = 30) provided positive feedback indicating the programme was easy to navigate, while 10.9% (*n* = 5) provided constructive feedback (e.g. need to include a table of contents, having titles for each session and making the tutorial more straightforward). For valuable, 84.8% of participants (*n* = 39) provided positive comments indicating that the programme helped them address their sleep needs, while only 2.2% of participants (*n* = 1) noted the programme did not have enough new information compared to what they already knew about sleep. For accessibility, positive comments were provided by 84.8% (*n* = 39) participants and constructive feedback by 10.9% (*n* = 5) of participants; comments indicated the programme was easy to understand and navigate, but a few participants requested captions and simpler terminology. For desirability, 71.7% of participants (*n* = 33) provided positive comments, while 15.2% (*n* = 7) provided constructive comments. Positive comments focused on the relevance of the information and that the programme was visually appealing. Constructive feedback focused on changes to presentation of information such as fewer videos, different fonts and adding/removing information; however, there was little consistency with this feedback as to what specifically needed to change.

Two dimensions had a balance of positive and constructive comments. For credibility, 47.8% of participants (*n* = 22) provided positive comments while 17.4% of participants (*n* = 8) provided constructive feedback, all of which was to provide more citations. For usability, positive feedback and constructive feedback were provided by 47.8% of participants (*n* = 22) each. There was differing opinions about the length of the programme with about half of the comments indicating it was a good length and half indicating it was too long. Constructive feedback also focused on the programme needing to be more mobile-friendly (see Supplementary Material for example feedback from participants).

No dimension had more negative than positive feedback. The favourite components of the programme were techniques for improving their sleep (*n* = 31 comments) and least favourite component was that the programme involved too much reading (*n* = 11 comments).

**Table 3.** Examples of qualitative feedback.

User experience dimension	Positive feedback	Constructive feedback	Percentage of participants with positive/constructive feedback
Credible	‘I could tell that this program was well-researched and came from a reputable source. It is from a university, which makes me comfortable. Additionally, it uses references (like in session 4 ‘Resources’) to supplement its own research.’	‘More references can be added to increase credibility’	47.8/17.4
Usable	‘The lessons were the perfect length! Usually I hate long slideshows/lessons that feel like university lectures, but this was perfect. I was engaged throughout.’	‘It takes too much time to complete the entire program.’	47.8/47.8
Findable	‘The material is organized and well located.’	‘The sessions were extremely easy to find, information within the lessons, not so much.’	65.2/10.9
Accessible	‘The information was presented in an easy-to-understand manner and a lot of the content was written in very simple language.’	‘The terminology is too long. It is relatively hard to understand for a simple topic.’	84.8/10.9
Valuable	‘Throughput the program I learned information that I didn’t know before, particularly when the program focused on something specific or on numbers. Unless people do research on their own about sleep I feel like this program fills in a lot of gaps. And because information gaps were filled in and applied to my original habits I was able to reach my goals (or make progress towards them).’	‘Not a ton of new info’	84.8/2.2
Useful	‘I felt that the program overall had good information that helped me understand and treat my sleep problems. I saw an improvement in my quality of sleep as I applied the suggestions from this program.’	No constructive feedback provided	73.9/0.0
Useful Session 4	‘I found it helpful that roadblocks were identified and possible ways to work around them were given.’	‘This is just a review session, I did not find it useful.’	41.3/50.0
Desirable	‘As for the content, I found that most of the lessons were informative and I ended with more information about sleep or a new plan to try. That made me want to come back and continue with the program.’	‘The plain text was a little annoying at times.’	71.7/15.0
Desirable Session 4	‘All the info provided was relevant to my goal of improving my sleep’	‘Not many images, only words. Therefore, it is not visually appealing.’	60.9/30.4

*Note:* Example quotations of qualitative feedback for the overall programme for each user experience dimension, and for the dimensions of useful and desirable from Session 4. Participants who did not provide qualitative feedback, or provided nondescript feedback (e.g. ‘good’, ‘fine’, ‘no suggestions’) were not included in the percentage of participants with positive/constructive feedback.

#### Session feedback ratings

##### Quantitative analyses

Of 28 mean usability ratings across four sessions and seven usability dimensions, no mean ratings were negative (score of 1 or 2). Neutral ratings (score of 3) were identified only for Session 4 on the user experience dimensions of useful (3.78) and desirable (3.43). The 26 remaining ratings were identified as positive (4–5), ranging from 4.0–4.46 (see Table 4).

**Table 4.** Session feedback ratings.

	Session 1 (*n *= 46)	Session 2 (*n *= 46)	Session 3 (*n *= 46)	Session 4 (*n *= 46)
Useful	4.32 (0.70)	4.26 (0.46)	4.38 (0.45)	3.78 (0.84)
Usable	4.16 (0.48)	4.03 (0.65)	4.13 (0.63)	4.37 (0.48)
Findable	4.13 (0.72)	4.00 (0.91)	4.17 (0.78)	4.22 (0.76)
Desirable	4.34 (0.48)	4.27 (0.48)	4.40 (0.45)	3.43 (0.87)
Accessible	4.37 (0.51)	4.09 (0.62)	4.28 (0.64)	4.32 (0.45)
Credible	4.23 (0.58)	4.32 (0.40)	4.29 (0.55)	4.45 (0.44)
Valuable	4.07 (0.79)	4.25 (0.52)	4.38 (0.42)	4.02 (0.70)

*Note:* Rating scale from 1 to 5 (1 = strongly disagree; 3 = neither agree nor disagree; 5 = strongly agree). Dimensions were derived from Morville's User Experience Honeycomb (Morville & Sullenger, 2010).

##### Qualitative analyses

As quantitative ratings only differed for Session 4 on usefulness and desirability, qualitative feedback is described for just those dimensions. For the usefulness dimension of Session 4, participants endorsed both positive and constructive feedback. 41.3% of participants (*n* = 19) mentioned positive comments, mainly regarding the session being useful for treating sleep in special situations (e.g. when on vacation). Constructive feedback was provided by 50% (*n* = 23), with participants commenting that there was no new information as the session summarized what they had already learned. For the dimension of desirability, participants’ qualitative feedback was more constructive than positive, with 60.9% of participants discussing mostly that the session had no appealing features, such as videos, or interactive activities. In contrast, 30.4% (*n* = 14) of participants made positive comments about the relevance of material and the session's visual appeal.

#### Percentage in support of change

In addition to analysing the data for themes, a percentage in support of change was calculated for suggestions or criticisms within each session questionnaire and for the programme questionnaire. Only one suggestion met the 10% criteria for considering making a change to the programme. In Session 4, 26 participants (56.5% of the total sample) reported that there were no appealing features such as videos, pictures, or interactive components in the session and only 9 participants (19.6% of the sample) countered with comments that suggested that they thought the session was visually appealing. This led to a percentage in support of change of 36.9%.

#### Additional qualitative feedback

Other qualitative feedback was provided on favourite and least favourite features of the sessions, with most responses indicating features as the interactive images, quizzes and videos (*n* = 137 comments) as favoured and the least favourite feature being the amount of text/reading (*n* = 48 comments) for Sessions 1–3. For Session 4, favourite features were the length of the session and the extra resources (*n* = 10 and *n* = 8, respectively) and least favourite feature was generally that there were no interactive features and no new information (*n* = 14 and *n* = 9 comments, respectively).

## Discussion

The current study presents evidence that the *BNBD-Youth* can be extended to be used in the university population, with some minor modifications. The study offers usability data with a large number of participants (*n* = 46) providing opinions on each session of the *BNBD-Youth* programme, as well as the programme overall. Based on the quantitative data collected, usability ratings across all seven of Morville's^54^ user experience dimensions were positive for the overall programme. Ratings were similarly positive for each session on five of the seven usability dimensions, including usable, valuable, findable, credible and accessible. For the remaining two dimensions of desirable and useful, average ratings were positive apart from Session 4, which had scores that fell in the neutral range.

The qualitative data collected evaluating the programme overall, consisted mostly of positive comments in the areas of usefulness, value, findability, accessibility and desirability. In terms of the usability of the programme, the majority suggested that the programme was user-friendly; however, there were mixed reviews on length, with some suggesting that the length was reasonable and some suggesting the programme to be too long; however, this constructive feedback did not meet the 10% criterion cut-off to be considered to make a change to the length in programme. Similarly, the credibility of the programme had mixed reviews, with participants’ constructive comments suggesting that the programme could use more article citation, though the constructive feedback did not reach the criterion for considering change. Moving forward, it may be worthwhile to incorporate more citations to promote more credibility and to remind the user of why the programme is the length it is (e.g. importance of the information included in the programme).

Qualitative feedback for the programme sessions was mixed on the dimensions of usefulness and desirability for Session 4. While some participants provided positive feedback suggesting that the session was useful for treating sleep in special situations (e.g. sleeping when on vacation), other participants suggested that the session was just a summary of previous learnings. Although the bulk of Session 4 is mainly a summary of previous sessions, the session also encourages participants to reflect on their goals and progress since starting the programme and provides new information on roadblocks that can arise when trying to obtain adequate sleep (e.g. what to do on vacation, during daylight savings). Furthermore, Session 4 differed on desirability. As Session 4 was mainly a summary, it has no extra features (e.g. videos interactive activities) and feedback reflected this, with most constructive feedback suggesting that there was not enough relevant information in the session for it to be desirable, as well participants noting they wanted more features. Of these two themes, only the comments on lack of features in the session met the criterion of 10% to consider making a change to the session.

Overall, users experienced *BNBD-Youth* as credible, valuable, accessible, useful, desirable, usable and findable. Strengths of the programme were that participants found the content easy to understand and valuable for addressing their sleep needs. Furthermore, participants found the programme useful in understanding sleep in general, understanding their own sleep difficulties and how to treat their sleep issues. Apart from Session 4, there was a general trend of enjoying the programme's features, such as interactive activities, images and videos. The main challenge of the programme was that there simply was not enough features in Session 4 to be desirable to university students. In keeping with the user feedback accrued in this study, changes will be made to the programme moving forward. In Session 4, pictures will be added to the slides to break up the text content. Furthermore, a video may be added with a brief message of congratulations to increase the interactivity of the session.

The current results were based on the seven dimensions of the User Experience Honeycomb framework (usability, usefulness, value, accessibility, credibility, findability and desirability). Other studies have discussed additional theoretical underpinnings of the intention to use mobile health services. One widely used framework, the technology acceptance model (TAM)^63^ suggests two key factors that influence users’ attitudes and behavioural intentions for using mobile health services: perceived usefulness and perceived ease of use. These factors are encompassed under the User Experience Honeycomb, under the usability and usefulness dimensions. On the contrary, Mouloudj et al. (2023) provided evidence for an extended Technology Acceptance model (TAM) by incorporating the concepts of perceived self-efficacy, attitudes towards digital applications and trust in the digital health system.^64^ While the User Experience Honeycomb does take into account trust (credibility), perceived self-efficacy and attitudes towards digital applications are additional factors that are not considered within the framework used and may be important to consider in the future. Additionally, the unified theory of acceptance and use of technology (UTAUT-2) model has been used to investigate intentions of using mobile healthcare apps in China.^65^ This model suggests seven factors that influence the acceptance and use of technology; performance expectancy (perception of how much the system will help an individual attain gains), effort expectancy (ease of use), social influence (the degree to which an individual feels it is important for others to believe he/she should use the system), facilitating conditions (the degree to which an individual believes that organizational and technical infrastructure exists to support use of the system), perceived risk, price value and perceived trust of the system. The User Experience Honeycomb takes into account many of these factors, including performance expectancy (desirability, value), effort expectancy (usability), facilitating conditions (accessibility, findability) and perceived trust (credibility). In the future it may be prudent to include social influence, perceived risk and price value, to bolster the likelihood of use of the programme.

Comparing the results of this study to prior BNBD-Youth studies, results were similar. The prior usability study with younger adolescents demonstrated mostly positive quantitative ratings with the exception of the dimension of useful and desirable for Sessions 1, 2 and 4 and value for Session 2 which all were in the neutral range, whereas these were positive for university students. In terms of meeting the 10% criterion to consider making changes to the programme, both studies found that for the dimension of desirability, in particular to add more interactive features, was met for change. However, in the study with younger adolescents there were additional suggestions including having fewer paragraphs and improved visual design and to increase compatibility of features and videos with varying media devices in the dimension of accessibility. These results would indicate that most usability issues were addressed after the study with younger adolescents, but some remain to be addressed further.

In comparing *BNBD-Youth* to other programmes more broadly, Digdon and Koble^42^ and Jones et al.'s^32^ interventions are similar in that both are passive interventions, delivering education about sleep via email and text message, respectively. In contrast, *BNBD-Youth* is an active intervention that includes psychoeducation and individual tailoring with interactive features such as drag-and-drop activities, quizzes and videos. Furthermore, in terms of usability, the results of the current study demonstrated that for the majority of participants, the interactive features were considered the favourite component of the intervention. Moreover, Digdon and Koble's study only used feedback to guide future iterations of the programme, once the programme was completed and only asked for participants opinions on the effectiveness of the strategies and what could be changed, but not on the actual email delivery as a method of intervention. Although Jones and colleagues’ programme used focus groups to inform the text message content, they did not discuss the results of this process. Compared to these studies, the *BNBD-Youth* study highlights the importance of systematically analysing usability data and reporting the results within the programme development process, to ensure appropriate modifications will be made that will impact the usability of the programme (e.g. adding to the desirability of the *BNBD-Youth* programme).

Similar to Fucito et al.,^30,31^ the *BNBD-Youth* programme incorporated user feedback in both the development of the programme itself and to evaluate usability once the programme was created. Unlike *BNBD-Youth*, Fucito and colleagues, only showed a PowerPoint of the programme to collect usability data and did not have students use the programme in real time. Furthermore, researchers did not report on usability data after beta-testing and as such, although focus groups were reported, it is unknown what the results of the beta-testing produced. Although modifications to the programme were reported, it could have been useful for the programme to report more specifically on the usability data to inform the development and implementation of usability studies more generally in the future, as well as to inform researchers on student likes and dislikes for creating sleep-related interventions for university students in the future. Finally, the BNBD-*Youth* intervention can be differentiated from Fucito et al., by its content, with Fucito's intervention addressing both sleep and alcohol use, whereas BNBD-*Youth* focuses strictly on sleep intervention, an issue that often appears on its own, without heavy alcohol use. This demonstrates the importance of BNBD-*Youth* in targeting students who have issues solely related to sleep. Finally, in terms of effectiveness of the programmes themselves, the programmes by Digdon and Koble^42^ and Fucito et al.^31^ were both found to improve sleep outcomes, while the programme by Jones and colleagues demonstrated no significant differences on sleep outcomes. Although *BNBD-Youth* has yet to be tested for effectiveness, the current study ensures that problems with usability will not interfere with its potential effectiveness.

Much like other eHealth sleep programmes, the *BNBD-Youth* programme aims to improve sleep health by providing psychoeducation and using evidence-based behavioural strategies. In contrast, more comprehensive programmes exist that target a multitude of sleep and mental health problems in one programme. One example of a more comprehensive health programme is The Transdiagnostic Intervention for Sleep and Circadian Dysfunction (TransS-C).^66^ The TransS-C treats a range of sleep and circadian rhythm issues across a range of mental disorders, by treating the common mechanisms that drive the comorbid disorders. The TransS-C programme incorporates the same behavioural sleep strategies, but additionally includes improving daytime functioning through healthy strategies to improve energy levels, coping with unhelpful beliefs about sleep and planning for how to maintain the healthy behaviour changes made during treatment, with seven additional optional modules that address other less common issues, depending on individual goals and needs. Some advantages to using a trans-diagnostic approach is that this approach can treat multiple disorders simultaneously and treating both can lead to reciprocal improvement in comorbid disorders. In contrast, the advantage of *BNBD-Youth* may be that individuals have less areas of improvement to focus on, which may lead to less feelings of being overwhelmed at the material. Additionally, fewer strategies to learn may lead to more self-efficacy, which is a factor that contributes to behaviour intention to use eHealth. It will be important to empirically test the advantages and disadvantages of comprehensive versus specific-focused programmes in upcoming research.

In summary, this study found that the *BNBD-Youth* programme is has high usability ratings by both younger and older adolescents and only a few minor modifications need to be made to the programme before moving ahead with further evaluation of the programme within the university student population. Furthermore, the results underscore the importance of utilizing user-centre designs in every stage of programme development in order to enhance usability and effectiveness. Beginning at the conception of *BNBD-Youth* (prior to including university students), information was gathered from youth on their preferences and desires, which were incorporated into the programme (e.g. using individual tailoring). Ultimately, developing a programme using a user-centred approach led to the creation of a programme rated positively on all user experience dimensions for university students, with only one change required in one of four sessions. Furthermore, as the current study sought to gain feedback to refine the programme, the feedback can now be incorporated into the programme before effectiveness testing, ensuring that the usability of the programme does not deter students from engaging in the programme in the future. Looking ahead, upon making the suggested modifications, *BNBD-Youth* appears to be a promising programme to use with university students.

### Strengths and limitations

A strength of this study was the relatively large sample of 46 students, with usability studies suggesting appropriate sample sizes ranging from 5 to 20 participants to catch 95% usability problems.^67^ Additionally, this study utilized a diverse sample of students from universities across Canada, including international students. Moreover, this study was conceptualized using a user-centre designed, capitalizing on end-users’ suggestions for what they wanted to see in the programme, what they enjoyed about the programme once developed and what aspects require modifications. Furthermore, this programme used a formal usability framework to evaluate usability perceptions, unlike the other programmes that incorporated user feedback into their designs.

A limitation of the study was that individuals self-selected to participate. As such, there is the possibility of selection bias, in which the sample collected may not reflect the overall population of interest, but a proportion of the population that was interested in participating in the research study. Another limitation is that honoraria were given for completing the questionnaires and as such, there was a potential external motivation to complete the programme and perhaps provide favourable ratings. Furthermore, as online questionnaires were used to collect qualitative data, some participants did not provide rich feedback (e.g. only ‘it was useful’ or ‘I liked the programme’). Another limitation of this study is that only self-report measures were used to gather feedback. As such, it is possible that students provided inaccurate feedback. An additional limitation of this study is the large percentage of attrition. After participants were enrolled in the programme, 37% of participants did not complete the study requirements. Although this percentage is common in online higher education^68–70^ there is a possibility that a proportion of these participants disliked the programme and thus did not continue with it, or did not wish to fulfil the demands of the study itself. Another limitation of the study is that session questionnaires were sent to participants at the beginning of each session (to allow for participants to complete the questionnaire while going through the session). As such, there is the possibility that participants completed the questionnaire before completing the session and thus their responses may not reflect their true experiences using the programme. Another limitation of the study is the disproportionate number of students who described themselves as international students (43%). In the 2019/2020 school year, Statistics Canada reported that international students made up approximately 20.6% of enrolments in Canadian universities.^71^ This suggests that the study may not generalize completely to a Canadian university student population. It should also be noted that this programme has been tested only with students in universities and so it does not generalize to the whole young adult student population (e.g. students who attend community colleges, vocational programmes and other non-traditional educational programmes). An additional limitation of the *BNBD-Uni* programme and the usability study, is that at the present, there are no described video options, closed captions or read-aloud functions within the programme, and as such, the programme is not fully accessible to users with auditory or visual impairments, and users who reported these impairments were excluded from the study. In the future, it will be beneficial to incorporate these functions into the *BNBD-Uni* programme, to ensure wider accessibility of the programme.

### Future directions

After incorporating the suggested changes to the *BNBD-Youth* programme to create the *BNBD-Uni* programme, it would be helpful to test the usability of the programme again with a larger sample size of university students, to ensure the generalization of findings to a wider range of university students. Furthermore, as other studies have evaluated usability using multiple methods (e.g. think-aloud activities, semi-structured interview), it would be prudent to expand the usability assessment by including other methods of evaluation to ensure detailed and accurate feedback. The next step after will be to complete effectiveness testing of the *BNBD-Uni* programme. A pre-post design, followed by a RCT testing sleep quality and quantity variables (e.g. objective measures of sleep using actigraphy data, subjective measures of sleep and sleep habits, including sleep diaries, sleep habits, quantity and quality questionnaires) before and after using the programme is warranted to assess the effectiveness of the programme in improving students’ sleep. Another avenue to consider in the future is to expand testing of the programme to additional student populations (e.g. college students, students in vocational programmes) and students with additional sleep concerns (e.g. poor sleep quality), to further assess the usability and effectiveness of the programme.

## Conclusions

*BNBD* – *Youth* is an eHealth sleep education programme that has high usability ratings both in a younger and older adolescent population and with minor modifications the programme is ready for further testing with university students. Quantitative and qualitative feedback from participants regarding the programme was generally positive. After tallying comments within both dimensions, the one area of feedback that made criteria for consideration of change was that participants wanted to see more images, activities and videos in Session 4. Taken together, the feedback from participants suggests that the programme is largely ready for rigorous effectiveness testing, the next stage in the development process. These results also demonstrate that it is possible to extend eHealth programmes to other ages with appropriate usability testing. Using the feedback from this study, the *BNBD-Uni* programme will be created and further tested.

## Supplemental Material

sj-docx-1-dhj-10.1177_20552076241260480 - Supplemental material for Usability of an eHealth sleep education intervention for university students Supplemental material, sj-docx-1-dhj-10.1177_20552076241260480 for Usability of an eHealth sleep education intervention for university students by Lindsay Rosenberg, Gabrielle Rigney, Anastasija Jemcov and Derek van Voorst, Penny Corkum in DIGITAL HEALTH

sj-docx-2-dhj-10.1177_20552076241260480 - Supplemental material for Usability of an eHealth sleep education intervention for university students Supplemental material, sj-docx-2-dhj-10.1177_20552076241260480 for Usability of an eHealth sleep education intervention for university students by Lindsay Rosenberg, Gabrielle Rigney, Anastasija Jemcov and Derek van Voorst, Penny Corkum in DIGITAL HEALTH

sj-docx-3-dhj-10.1177_20552076241260480 - Supplemental material for Usability of an eHealth sleep education intervention for university students Supplemental material, sj-docx-3-dhj-10.1177_20552076241260480 for Usability of an eHealth sleep education intervention for university students by Lindsay Rosenberg, Gabrielle Rigney, Anastasija Jemcov and Derek van Voorst, Penny Corkum in DIGITAL HEALTH
