# Supplementary material for: Usability of an eHealth sleep education intervention for university students
Source: Digit Health. 2024 Jun 5;10:20552076241260480. doi: 10.1177/20552076241260480 (PMC11155311; doi:10.1177/20552076241260480)
Supplement: sj-docx-1-dhj-10.1177_20552076241260480 - Supplemental material for Usability of an eHealth sleep education intervention for university students [file sj-docx-1-dhj-10.1177_20552076241260480.docx]

SESSION FEEDBACK QUESTIONNAIRE

**QUESTIONNAIRE PRESENTED AFTER EACH SESSION**

Well done on making it through this *BNBD-Youth* section!

The following questions will ask you about your experience with the section. Please answer the questions as openly and honestly as possible. Your feedback will help with modifications and updates to the final version of the online program.

This questionnaire will take you approximately 15 minutes to complete.

[*THE QUESTIONS FOR TOPICS 1-9 WERE PRESENTED WITH EVERY BNBD-YOUTH SESSION. THE QUESTIONS FOR TOPIC 10 WERE SPECIFIC TO THE FEATURES PRESENTED WITHIN EACH SESSION*]

1. **Useful**

- This session provided information that helped me better *understand* my sleep problems.
  - Strongly agree
  - Agree
  - Neither agree or disagree
  - Disagree
  - Strongly disagree
- This session provided information that would help me *treat* my sleep problems.
  - Strongly agree
  - Agree
  - Neither agree or disagree
  - Disagree
  - Strongly disagree

Please provide comments that support your rating about the **usefulness** of this session in helping to understand and/or treat your sleep problems. Include any suggestions you may have to make this program more **useful:** [text box]

1. **Usable**

- This session was user-friendly (easy-to-use).
  - Strongly agree
  - Agree
  - Neither agree or disagree
  - Disagree
  - Strongly disagree
- This session took a reasonable amount of time for me to complete.
  - Strongly agree
  - Agree
  - Neither agree or disagree
  - Disagree
  - Strongly disagree

Please provide comments that support your rating about the **usability** of this session (user-friendly, quick to complete). Include any suggestions you may have to improve **usability**: [text box]

1. **Findable**

- Information/content within the overall *session* was easy to find.
  - Strongly agree
  - Agree
  - Neither agree or disagree
  - Disagree
  - Strongly disagree
- Information/content within each of the individual *lessons* within the session was easy to find.
  - Strongly agree
  - Agree
  - Neither agree or disagree
  - Disagree
  - Strongly disagree

Please provide comments that support your rating about the how **findable** (easy to find information, organized lessons) this session was. Include any suggestions you may have to improve **findability**: [text box]

1. **Desirable**

- This session contained information that I wanted to learn about.
  - Strongly agree
  - Agree
  - Neither agree or disagree
  - Disagree
  - Strongly disagree
- This session was visually appealing (think about the color scheme, font style, font size, and graphics).
  - Strongly agree
  - Agree
  - Neither agree or disagree
  - Disagree
  - Strongly disagree

Please provide comments that support your rating about the **desirability** of this section (desired information, visually appealing). Include any suggestions you may have to improve **desirability**: [text box]

1. **Accessible**

- It was easy to navigate this session.
  - Strongly agree
  - Agree
  - Neither agree or disagree
  - Disagree
  - Strongly disagree
- The information provided in this session was easy for me to understand.
  - Strongly agree
  - Agree
  - Neither agree or disagree
  - Disagree
  - Strongly disagree

Please provide comments that support your rating about the **accessibility** (easy to navigate and understand) of this section. Include any suggestions you may have to improve **accessibility**: [text box]

1. **Credible**

- Overall, I believe that the information provided in this session comes from a reputable source.
  - Strongly agree
  - Agree
  - Neither agree or disagree
  - Disagree
  - Strongly disagree
- I trust the information from this session enough to use it to try and treat my sleep problems.
  - Strongly agree
  - Agree
  - Neither agree or disagree
  - Disagree
  - Strongly disagree

Please provide comments that support your rating about the **credibility** of this intervention (trusting the information, information coming from credible source) Include any suggestions you may have that would help make the information appear more **credible**: [text box]

1. **Valuable**

- Overall, the information provided in this session helped me reach my goals for participating in this program (e.g., learning about sleep, managing sleep).
  - Strongly agree
  - Agree
  - Neither agree or disagree
  - Disagree
  - Strongly disagree
- Overall, I have learned information that I did not know before participating in this program.
  - Strongly agree
  - Agree
  - Neither agree or disagree
  - Disagree
  - Strongly disagree

Please provide comments that support your rating about the **value** of this intervention (e.g., moving toward goals, learning new information). Include any suggestions you may have to improve the **value** of the section: [text box]

1. **Features**

*Features are any of the activities that you used throughout the session. This includes quizzes, videos, drag and drop or question & answer, interactive images or diagrams and text.*

- I would refer back to this session at a later time while having access to this program.
  - Strongly agree
  - Agree
  - Neither agree or disagree
  - Disagree
  - Strongly disagree
- What were your favorite feature(s) from this session?
  - [text box]
- What were your least favorite feature(s) from this session?
  - [text box]
- What features(s) were most engaging from this session?
  - [text box]
- What feature(s) were least engaging from this session?
  - [text box]
- What did you learn the most from this session?
  - [text box]

***Session 1: What is Sleep?***

- The “Are you a Lark or an Owl” quiz helped me to better understand my natural sleep timing.
  - Strongly agree
  - Agree
  - Neither agree or disagree
  - Disagree
  - Strongly disagree

***Session 2: Your sleep***

- Reviewing my *sleep diary data* in this session helped me to understand my sleep and identify problem areas.
  - Strongly agree
  - Agree
  - Neither agree or disagree
  - Disagree
  - Strongly disagree
- The *sleep hygiene questionnaire* helped me to understand my sleep and identify problem areas.
  - Strongly agree
  - Agree
  - Neither agree or disagree
  - Disagree
  - Strongly disagree
- The *sleep quality questionnaire* helped me to understand my sleep and identify problem areas.
  - Strongly agree
  - Agree
  - Neither agree or disagree
  - Disagree
  - Strongly disagree

**Session 3: Healthy sleep practices**

- The length of time between sessions 1-2 and session 3 was appropriate
  - Strongly agree
  - Agree
  - Neither agree or disagree
  - Disagree
  - Strongly disagree
- Please provide any comments about the length of time between sessions (e.g., it too long/ not long enough?)
  - [text box]
- Did you complete the diary between sessions?
  - Yes
  - No
- Being required to complete the diary before moving to the next session would have turned me away from the program.
  - Strongly agree
  - Agree
  - Neither agree or disagree
  - Disagree
  - Strongly disagree
- Receiving *personalized feedback* for each of the ABCs of SLEEPING was helpful.
  - Strongly agree
  - Agree
  - Neither agree or disagree
  - Disagree
  - Strongly disagree
- Developing a *sleep plan* based on the personalized feedback of the ABCs of SLEEPING was helpful.
  - Strongly agree
  - Agree
  - Neither agree or disagree
  - Disagree
  - Strongly disagree
- I plan on using my personalized sleep plan to address my sleep problems.
  - Strongly agree
  - Agree
  - Neither agree or disagree
  - Disagree
  - Strongly disagree

**Session 4: Checking in**

- Did you complete the diary between sessions?
  - Yes
  - No
  - [text box]

1. **General Feedback**

- Overall, I was satisfied with this session.
  - Strongly agree
  - Agree
  - Neither agree or disagree
  - Disagree
  - Strongly disagree
- This session is ready to be used by university students experiencing sleep problems.
  - Strongly agree
  - Agree
  - Neither agree or disagree
  - Disagree
  - Strongly disagree
- Do you think anything should be *added* to this session for it to better meet your needs?
  - Yes
  - Maybe
  - No
    - If Yes, please provide your suggestions [text box]
- Do you think anything should be *deleted* from this session for it to better meet your needs?
  - Yes
  - Maybe
  - No
    - If Yes, please provide your suggestions [text box]
- Do you think anything in this session should be *changed or reordered* for it to better meet your needs?
  - Yes
  - Maybe
  - No
    - If Yes, please provide your suggestions [text box]

Please provide any additional feedback you have about the section. If you do not believe it is **ready for use**, in what ways must it be modified to be ready? [text box]
