# Supplementary material for: Usability of an eHealth sleep education intervention for university students
Source: Digit Health. 2024 Jun 5;10:20552076241260480. doi: 10.1177/20552076241260480 (PMC11155311; doi:10.1177/20552076241260480)
Supplement: sj-docx-2-dhj-10.1177_20552076241260480 - Supplemental material for Usability of an eHealth sleep education intervention for university students [file sj-docx-2-dhj-10.1177_20552076241260480.docx]

PROGRAM FEEDBACK QUESTIONNAIRE

Well done on making it through the full program!

The following questions will ask you about your overall impressions of the *Better Nights, Better Days – Youth (BNBD-Youth)* program. Please answer the questions as openly and honestly as possible. Your feedback will be used for modifications and updates to the final version of the online program to make it useable for university students.

Below are a series of statements that relate to your impressions of the *BNBD-Youth* program OVERALL. Please indicate your agreement with each statement and then provide comments to explain your ratings within each category.

This questionnaire will take you approximately 25 minutes to complete.

1. **Useful**

- Overall, *BNBD-Youth* provided information that helped me better *understand* my sleep problems.
  - Strongly agree
  - Agree
  - Neither agree or disagree
  - Disagree
  - Strongly disagree
- Overall, *BNBD-Youth* provided information that would help me *treat* my sleep problems.
  - Strongly agree
  - Agree
  - Neither agree or disagree
  - Disagree
  - Strongly disagree

Please provide comments that support your rating about the **usefulness** of the *BNBD-Youth* program in helping to understand and/or treat your sleep problems. Include any suggestions you may have to make this program more **useful:** [text box]

1. **Usable**

- Overall, I found *BNBD-Youth* to be user-friendly (easy-to-use).
  - Strongly agree
  - Agree
  - Neither agree or disagree
  - Disagree
  - Strongly disagree
- Overall, *BNBD-Youth* took a reasonable amount of time for me to complete.
  - Strongly agree
  - Agree
  - Neither agree or disagree
  - Disagree
  - Strongly disagree

Please provide comments that support your rating about the **usability** of the *BNBD-Youth* program (user-friendly, quick to complete). Include any suggestions you may have to improve **usability**: [text box]

1. **Findable**

- Information/content was easy to find within *BNBD-Youth*.
  - Strongly agree
  - Agree
  - Neither agree or disagree
  - Disagree
  - Strongly disagree
- Features (e.g., daily diary) were easy to find within *BNBD-Youth*.
  - Strongly agree
  - Agree
  - Neither agree or disagree
  - Disagree
  - Strongly disagree

Please provide comments that support your rating about the how **findable** (easy to find information, organized lessons) *BNBD-Youth* was. Include any suggestions you may have to improve **findability**: [text box]

1. **Desirable**

- Overall, *BNBD-Youth* contained information that I wanted to learn about.
  - Strongly agree
  - Agree
  - Neither agree or disagree
  - Disagree
  - Strongly disagree
- Overall, *BNBD-Youth* was visually appealing (think about the color scheme, font style, font size, and graphics).
  - Strongly agree
  - Agree
  - Neither agree or disagree
  - Disagree
  - Strongly disagree

Please provide comments that support your rating about the **desirability** of the *BNBD-Youth* program (desired information, visually appealing). Include any suggestions you may have to improve **desirability**: [text box]

1. **Accessible**

- It was easy to navigate *BNBD-Youth*.
  - Strongly agree
  - Agree
  - Neither agree or disagree
  - Disagree
  - Strongly disagree
- Overall, the information provided in *BNBD-Youth* was easy for me to understand.
  - Strongly agree
  - Agree
  - Neither agree or disagree
  - Disagree
  - Strongly disagree

Please provide comments that support your rating about the **accessibility** of the *BNBD-Youth* program (easy to navigate and understand, technology used). Include any suggestions you may have to improve **accessibility**: [text box]

1. **Credible**

- Overall, I believe that the information provided in *BNBD-Youth* comes from a reputable source.
  - Strongly agree
  - Agree
  - Neither agree or disagree
  - Disagree
  - Strongly disagree
- I trust the information from *BNBD-Youth* enough to feel comfortable using it to try and treat my sleep problems.
  - Strongly agree
  - Agree
  - Neither agree or disagree
  - Disagree
  - Strongly disagree

Please provide comments that support your rating about the **credibility** of the *BNBD-Youth* program. Include any suggestions you may have that would help make the information appear more **credible**: [text box]

1. **Valuable**

- Overall, the information provided by *BNBD-Youth* helped me reach my goals for participating in this program (e.g. learning about sleep, managing sleep).
  - Strongly agree
  - Agree
  - Neither agree or disagree
  - Disagree
  - Strongly disagree
- Overall, I have learned information that I did not know before participating in this program.
  - Strongly agree
  - Agree
  - Neither agree or disagree
  - Disagree
  - Strongly disagree

Please provide comments that support your rating about the **value** of the *BNBD-Youth* program (e.g., moving toward goals, learning new information). Include any suggestions you may have to improve the **value** of the program: [text box]

1. **Features**

*Features are any of the activities that you used throughout the session. This includes quizzes, videos, drag and drop or question & answer, interactive images or diagrams and text*

- Overall, I liked the interactive features (e.g., drag and drop, interactive images, presentation of text) in *BNBD-Youth*.
  - Strongly agree
  - Agree
  - Neither agree or disagree
  - Disagree
  - Strongly disagree
- I used the Notes feature within *BNBD-Youth*.
  - Yes
  - No
- Overall, I liked the sleep diary integrated in the *BNBD-Youth* program *(Note: This question is NOT referring to the diary that you filled out at the beginning and end of your participation in this research study*).
  - Strongly agree
  - Agree
  - Neither agree or disagree
  - Disagree
  - Strongly disagree
- Overall, the reminders to complete the sleep diary were helpful.
  - Strongly agree
  - Agree
  - Neither agree or disagree
  - Disagree
  - Strongly disagree
- Overall, I think the number of sleep diary reminders was appropriate.
  - Strongly agree
  - Agree
  - Neither agree or disagree
  - Disagree
  - Strongly disagree
- Overall, I liked the videos in *BNBD-Youth*.
  - Strongly agree
  - Agree
  - Neither agree or disagree
  - Disagree
  - Strongly disagree
- Overall, I think the videos added educational value to *BNBD-Youth*.
  - Strongly agree
  - Agree
  - Neither agree or disagree
  - Disagree
  - Strongly disagree

Please provide comments that support your rating about the **features** of *BNBD-Youth*. Include any suggestions you may to improve the **features** included in the program: [text box]

1. **Technology**

- Overall, the *BNBD-Youth* program was accessible from my chosen device(s) when and where desired.
  - Strongly agree
  - Agree
  - Neither agree or disagree
  - Disagree
  - Strongly disagree

*The following questions ask about your experience accessing BNBD-Youth on your TECHNOLOGY HARDWARE. Please select the response below that best describes the technology you used the most.*

- What type(s) of hardware did you typically use? [Select multiple options]
  - Desktop
  - Laptop
  - Tablet
  - Smartphone
  - Other [text box]
- What type(s) of OS system(s) were you typically using? [Select multiple options]
  - Windows
  - MAC
  - Google (specific to smartphones)
  - Other [text box]
- Do you think anything should be added to this program for it to better meet your needs? If ‘yes’ please provide your suggestions
  - Yes
  - No
  - [text box]
- What internet browser(s) were you typically using? [Select multiple options]
  - Firefox
  - Google Chrome
  - Internet Explorer
  - Microsoft Edge
  - Other [text box]
- On average, how quickly did the site load?
  - 1 second
  - 2 seconds
  - 3 seconds
  - 4 seconds
  - 5 seconds
  - More than 5 seconds
- How often did the site crash?
  - Never
  - 1-2 times
  - 3-4 times
  - 4-5 times
  - More than 5 times

If it did crash, what was happening when it did? [text box]

1. **General Feedback**

- Overall, I was satisfied with *BNBD-Youth*.
  - Strongly agree
  - Agree
  - Neither agree or disagree
  - Disagree
  - Strongly disagree
- Overall, the way in which the information was presented was satisfactory (e.g. text, videos, interactive activities)
  - Strongly agree
  - Agree
  - Neither agree or disagree
  - Disagree
  - Strongly disagree
- Overall, I liked how the label “Sessions” was used to describe each of the main components of *BNBD-Youth*.
  - Strongly agree
  - Agree
  - Neither agree or disagree
  - Disagree
  - Strongly disagree
    - If Disagree or Strongly disagree, please provide your suggestions for a different label to describe the main components of *BNBD-Youth*. [text box]
- Overall, I liked how the label “Lessons” was used to describe the components within each of the sessions.
  - Strongly agree
  - Agree
  - Neither agree or disagree
  - Disagree
  - Strongly disagree
- *BNBD-Youth* is ready to be used by adolescents/young adults experiencing sleep problems.
  - Strongly agree
  - Agree
  - Neither agree or disagree
  - Disagree
  - Strongly disagree
- Everything I expected to be included in the *BNBD-Youth* program was there.
  - Strongly agree
  - Agree
  - Neither agree or disagree
  - Disagree
  - Strongly disagree
- More information/materials need to be added to *BNBD-Youth* to better meet my needs.
  - Strongly agree
  - Agree
  - Neither agree or disagree
  - Disagree
  - Strongly disagree
    - If Agree or Strongly Agree, please provide your suggestions [text box]
- Some information/material should be *deleted* from *BNBD-Youth* for it to better meet my needs.
  - Strongly agree
  - Agree
  - Neither agree or disagree
  - Disagree
  - Strongly disagree
    - If Agree or Strongly Agree, please provide your suggestions [text box]
- *BNBD-Youth* should be *changed or reordered* for it to better meet my needs.
  - Strongly agree
  - Agree
  - Neither agree or disagree
  - Disagree
  - Strongly disagree
    - If Agree or Strongly Agree, please provide your suggestions [text box]
- I would recommend this program to university students for their sleep problems.
  - Strongly agree
  - Agree
  - Neither agree or disagree
  - Disagree
  - Strongly disagree
    - If Disagree or Strongly disagree, why not? [text box]
    - If Agree or Strongly agree, please provide any comments or suggestions that you would like to pass along to future university students who will be using this program. [text box]
- Would you change anything about this program to make it more relevant for university students with insomnia symptoms?
  - Yes
    - If yes, please provide what you would change? [text box]
  - Maybe
  - No

Please provide comments that support your rating of the *BNBD-Youth* program ***overall.***

Include any suggestions you may have to improve *BNBD-Youth* **overall**:

What are the components that you liked the least about *BNBD-Youth*?

What was the most valuable or helpful thing you learned from *BNBD-Youth*?

**We are appreciative of your feedback.**
