# Supplementary material for: Usability of an eHealth sleep education intervention for university students
Source: Digit Health. 2024 Jun 5;10:20552076241260480. doi: 10.1177/20552076241260480 (PMC11155311; doi:10.1177/20552076241260480)
Supplement: sj-docx-3-dhj-10.1177_20552076241260480 - Supplemental material for Usability of an eHealth sleep education intervention for university students [file sj-docx-3-dhj-10.1177_20552076241260480.docx]

***Overall Satisfaction and Readiness for Use***

|  | Session 1 | Session 2 | Session 3 | Session 4 | Overall Program |
| --- | --- | --- | --- | --- | --- |
| *I would refer back to this session at a later time while having access to this program.* | 3.82 (.89)  (n = 45) | 3.96 (.74)  (n = 45) | 4.17 (.68) | 3.67 (1.0)  (n = 45) | 4.35 (.48) |
| *Overall I was satisfied with this session/program.* | 4.02 (.65) | 4.22 (.51) | 4.43 (.50) | 3.89 (.74) | 4.29 (.51)  (n = 45) |
| *This session/program is* *ready to be used by adolescents/young adults experiencing sleep problems.* | 3.93 (.83) | 4.11 (.64) | 4.22 (.70) | 3.85 (.84) | 4.28 (.78) |
| *I would recommend this program to adolescents/young adults for their sleep problems.* | -- | -- | -- | -- | 4.07  (.88) |

Note: Questions based on a rating scale from 1-5 (1 = strongly disagree; 3 = neither agree nor disagree; 5 = strongly agree). All 46 participants responded to the questions unless otherwise stated.

***Content Amount and Order***

|  | Session 1 | Session 2 | Session 3 | Session 4 | Overall Program |
| --- | --- | --- | --- | --- | --- |
| *Do you think anything should be added to this session/program for it to better meet your needs?* | 2.67 (.70) | 2.91 (.35) | 2.85 (.52) | 2.80 (.58) | 2.35 (.80) |
| *Do you think anything should be deleted from this session/program for it to better meet your needs?* | 2.72 (.69) | 2.98 (.15) | 2.93 (.33) | 2.65 (.74) | 2.89 (1.1) |
|  |  |  |  |  |  |
| *Do you think anything in this session/program should be changed or reordered for it to better meet your needs?* | 2.82 (.58)  (n = 44) | 2.91 (.41) | 2.91 (.41) | 2.91 (.41) | 2.54 (.88) |

Note: For the Session Feedback Questionnaires questions were rated on a scale of 1-3 (1 = yes, 2 = maybe, 3 = no). For the Overall Program Feedback Questionnaire questions were rated on a scale from 1-5 (1 = strongly disagree; 3 = neither agree nor disagree; 5 = strongly agree). All 46 participants responded to the questions unless otherwise stated.
